# Identification of Acute Myeloid Leukemia Bone Marrow Circulating MicroRNAs

**DOI:** 10.3390/ijms21197065

**Published:** 2020-09-25

**Authors:** Douâa Moussa Agha, Redouane Rouas, Mehdi Najar, Fatima Bouhtit, Najib Naamane, Hussein Fayyad-Kazan, Dominique Bron, Nathalie Meuleman, Philippe Lewalle, Makram Merimi

**Affiliations:** 1 Laboratory of Experimental Hematology, Department of Haematology, Jules Bordet Institute, Université Libre de Bruxelles, 1000 Brussels, Belgium; douaa.moussa@gmail.com (D.M.A.); redouane.rouas@bordet.be (R.R.); bouhtitfatima@gmail.com (F.B.); hfayyadk@gmail.com (H.F.-K.); dominique.bron@bordet.be (D.B.); philippe.lewalle@bordet.be (P.L.); 2 Osteoarthritis Research Unit, University of Montreal Hospital Research Center (CRCHUM), Department of Medicine, University of Montreal, Montreal, QC H2X 0A9, Canada; mnajar@ulb.ac.be; 3 Genetics and Immune Cell Therapy Unit, Faculty of Sciences, University Mohammed Premier, Oujda 60000, Morocco; 4 Translational and Clinical Research Institute, Newcastle University, Newcastle upon Tyne NE2 4HH, UK; najib.naamane@newcastle.ac.uk; 5 Laboratory of Clinical Cell Therapy, Jules Bordet Institute, Université Libre de Bruxelles, 1070 Brussels, Belgium; nathalie.meuleman@bordet.be

**Keywords:** acute myeloid leukemia, bone marrow, biomarkers, circulating microRNAs, oncogene, tumor suppressor, immune regulation, tumor microenvironment

## Abstract

Background: In addition to their roles in different biological processes, microRNAs in the tumor microenvironment appear to be potential diagnostic and prognostic biomarkers for various malignant diseases, including acute myeloid leukemia (AML). To date, no screening of circulating miRNAs has been carried out in the bone marrow compartment of AML. Accordingly, we investigated the circulating miRNA profile in AML bone marrow at diagnosis (AMLD) and first complete remission post treatment (AMLPT) in comparison to healthy donors (HD). Methods: Circulating miRNAs were isolated from AML bone marrow aspirations, and a low-density TaqMan miRNA array was performed to identify deregulated miRNAs followed by quantitative RT-PCR to validate the results. Bioinformatic analysis was conducted to evaluate the diagnostic and prognostic accuracy of the highly and significantly identified deregulated miRNA(s) as potential candidate biomarker(s). Results: We found several deregulated miRNAs between the AMLD vs. HD vs. AMLPT groups, which were involved in tumor progression and immune suppression pathways. We also identified significant diagnostic and prognostic signatures with the ability to predict AML patient treatment response. Conclusions: This study provides a possible role of enriched circulating bone marrow miRNAs in the initiation and progression of AML and highlights new markers for prognosis and treatment monitoring.

## 1. Introduction

Acute myeloid leukemia (AML) is a common myelogenous malignancy in adults that is often characterized by disease relapse. It is caused by the acquisition of cytogenetic and molecular abnormalities by a hematopoietic stem cell, which transforms it into a leukemic stem cell that self-renews and proliferates [1]. Although many researchers are interested in improving our understanding of AML disease evolution [2], its pathogenesis, resistance to treatments and escape from immune surveillance have not yet been fully elucidated [3]. Despite recent advances in understanding the molecular basis of this cancer, allowing the development of target-specific therapies, more than 50% of patients with AML relapse and die [4]. They develop resistance to treatment caused by different genetic and immunosuppressive mechanisms. These mechanisms include changes in the expression levels of both intracellular and circulating microRNAs (miRNAs) [3,5,6,7]. Moreover, AML has been proven to be a heterogeneous disease, and to date, the cytogenetic classification fails to predict the clinical outcome for a large group of patients and therefore fails to guide the continuation of treatment in almost one-third of the patients [8]. The prognostic factors currently used remain unsatisfactory, and it is necessary to explore new biomarkers for the diagnosis, prognosis, and therapeutic targets of AML to develop more effective surveillance and treatment programs [3].

Small (19- to 22-nucleotide long) noncoding RNAs called microRNAs are crucial for the posttranscriptional regulation of gene expression. They play an integral role in numerous biological processes, including the immune response, cell-cycle control, metabolism, stem cell self-renewal and differentiation [9]. Aberrant miRNA expression is associated with many diseases, including cancer, and miRNA-based drugs represent a novel and potentially powerful therapeutic approach [10,11,12]. Several studies have proposed using miRNAs as biomarkers for diagnosis, prediction and prognosis in cancer diseases, including AML [13,14]. Extracellular miRNAs, namely, those circulating in peripheral blood, seem to be attracting increasing attention from researchers. Given the ease with which miRNAs can be isolated and their structural stability under different conditions of treatment and sample isolation, they are also proposed as diagnostic, prognostic or predictive biomarkers in several cancers [15,16].

In AML, peripheral blood circulating miRNAs have been detected and could be useful as noninvasive biomarkers for the detection of leukemia at the time of diagnosis and prognosis [3,7,14,17]. In our laboratory, we previously identified eight plasma miRs differentially expressed between healthy donors and AML patients, showing that miR-150 and miR-342 are downregulated in the plasma of AML patients at diagnosis, and their expression levels in complete remission AML patients resembled those in healthy controls [18]. Zhi et al. performed an analysis using AML serum samples and showed a significant increase in six miRNAs (miR-10a-5p, miR-93-5p, miR-129-5p, miR-155-5p, miR-181b-5p and miR-320d) [19]. The level of miR-181b-5p in serum, on the other hand, is significantly associated with an increased survival rate [19]. Yan J. and his team have shown that miR-217, a tumor suppressor, [17] is deregulated in patients with AML and more importantly in patients with low genetic risk. Other circulating miRNAs also seem of great interest for AML prognosis, namely, miR-210, whose deregulation was observed in patients in complete remission (CR) [20]. Patients expressing miR-210 have a significantly worse overall survival (OS), and those with weak expression of miR-328 have a shorter OS [20,21]. A significantly reduced expression of miR-638 has been shown in AML patients and increased in CR [22]. Another study carried out by Tian C et al. showed that the plasma expression of miR-192 is also increased in AML patients with favorable cytogenetic risk and high OS [23].

Several studies have focused on the profile of peripheral blood circulating miRNAs in acute myeloblastic leukemia. To date, no study has been published on miRNAs in the main microenvironment of AML development, namely, the bone marrow. It remains unknown how miRNAs released in the bone marrow niche in which AML arises are subverted to support leukemic cells and protect them from immunosurveillance. The contribution of the tumor microenvironment (TME) to the survival, spread, and relapse of AML in the bone marrow is crucial in the development, progression, immune response escape, and therapy failure in leukemia. Therefore, the aim of this study was to investigate, for the first time, whether circulating miRNAs display differential expression profiles in the bone marrow of AML patients at diagnosis and first complete remission after treatment in comparison with healthy donors. Such screening will highlight the crucial role of enriched circulating bone marrow miRNAs in AML diagnosis and prognosis and their impact on both disease progression and response to treatment.

## 2. Results

### 2.1. Bone Marrow Circulating miRNAs Expression Profile in Newly Diagnosed AML Patients

A few studies have focused on circulating peripheral blood miRNAs, but at this time, no studies have characterized them in the principal compartment of AML disease, namely, the bone marrow. We detected miRNA expression in bone marrow body fluid of AML patients at diagnosis (AMLD) (*n* = 27), and we compared their profile to that in healthy donors (HD) (*n* = 11). Moreover, miRNAs with a C*t* value > 35 in either the AML group or the control group were excluded from further analysis. Differentially expressed (DE) miRNAs between AMLD and HD samples, of which 61 were upregulated and 37 were downregulated, were identified with a threshold of *p* < 0.05 and a 1,5-fold change (Table 1).

**Figure 1:**
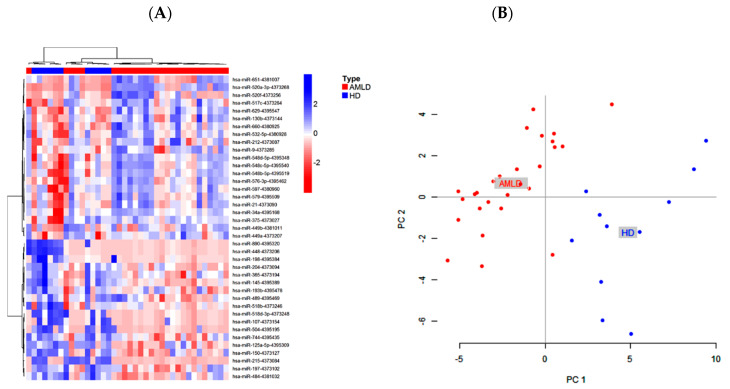
Differentially expressed bone marrow circulating miRNAs in newly diagnosed AML patients compared to healthy donors. (**A**) Hierarchical clustering and heatmap of microRNA (miRNA) expression profile in acute myeloid leukemia (AML) (*n* = 27) versus healthy donors (*n* = 11). The list of miRNAs was filtered with a *p*-value < 0.005 and fold change ≤ 0.5 or ≥ 2. A total of 39 miRNAs (21 up- and 18 downregulated) were significant differentially expressed. The color key indicates centred and standardized miRNA expression levels (Z-scores). Upper color bar labels samples by baseline diagnosis. (**B**) Principal Component Analysis (PCA) showing the grouping of AML patients (red) and HD (blue) based on the 39 DEmiRNAs profile.

The clustering of the pretreated AML patients and HD based on the highly significant DE miRNAs (21 up and 18 down *p* < 0.005 and 2 FC) showed a high separation between the two groups (Figure 1A,B). Interestingly, 16 of these DE miRNAs were undetectable or expressed at very low levels in one of the two groups (miR-520a-3p, miR-548d, miR-449b, miR-49-3p, miR-520f, miR-330-5p, and miR-34c-5p in HD patients and miR-326, miR-198, miR-miR-518d, miR-107, miR-215, miR-504, miR-890, and miR-448 in AML), suggesting their important role in the progression of AML disease.

To validate our data analysis, the levels of the top 46 differentially expressed miRNAs with very high statistical significance were quantified by RT-qPCR in a second independent cohort including 15 AML patient bone marrow aspiration samples. The results showed that 30 of the 46 miRNAs tested and identified as DE in the first cohort were confirmed in the second cohort (Table 2), while 14 were also DE in the same way as in the first cohort but were statistically insignificant. Only two miRNAs out of the 46 tested showed a different profile between the first and second cohorts, suggesting the need to increase the number of patients in our second cohort.

**Figure 2:**
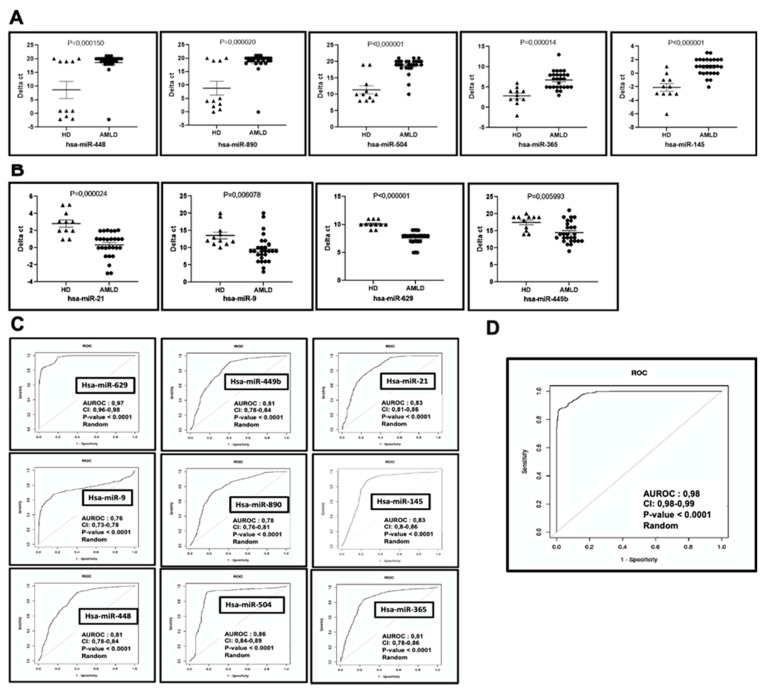
AML bone marrow circulating miRNA signature. The dot blot (**A**,**B**) and receiver operating characteristic (ROC) curves (**C**,**D**) reflecting the ability of the bone marrow circulating miRNA expression signature to differentiate the AML cases (*n* = 27) from the controls (*n* = 11). (**A**) Downregulated miRNAs: miR-448, 890, 504, 145, 365 and. (**B**) Upregulated miRNAs: miR-9; 21, 629 and 449b. (**C**) ROC curves reflecting the performance of the individual features of the 9 miRNA signature in discriminating AML patients from healthy donors. (**D**) Combined ROC showing the overall discriminatory power of the 9-miRNA signature. AUROC: Area Under ROC; CI: 95% Confidence Intervals; *p*-value: correspond to a Mann-Whitney U test addresses testing the null hypothesis of the AUROC is 0.5 (i.e., the classifier is random).

Our results permit us to establish an AML bone marrow circulating miRNA signature consisting of downregulated miR-448, miR-890, miR-504, miR-145, and miR-365 (Figure 2A) and upregulated miR-9, miR-21, miR-629 and miR-449b (Figure 2B), with highly significant AUCs (Figure 2C) and highly differentially expressed fold changes (*p*-value < 0.005; FC > 2). The combination of these miRNAs has diagnostic value, enabling the identification of AML with high sensitivity and specificity (AUC = 0.98; *p*-value < 0.0001) (Figure 2D).

**Figure 3:**
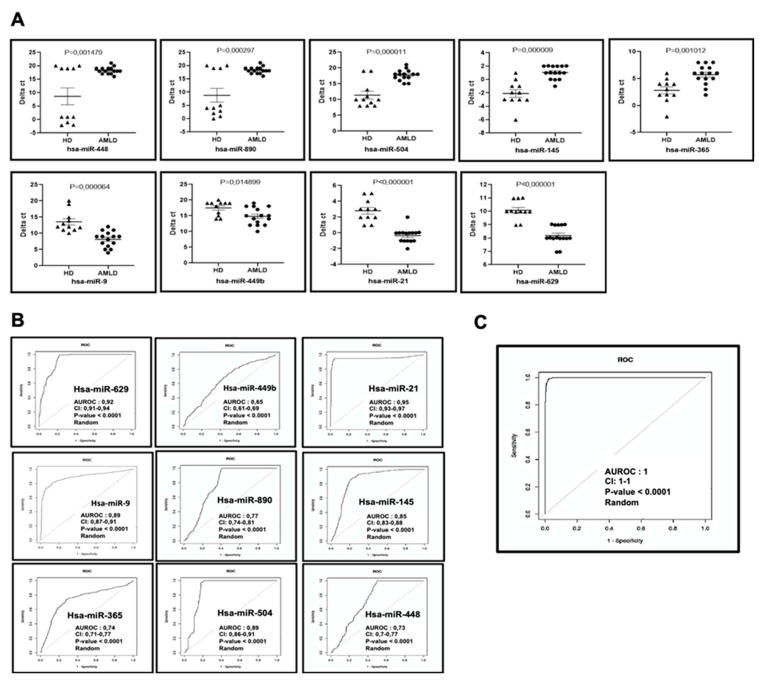
Confirmation of differentially expressed miRNAs in a second cohort (*n* = 15).(**A**) The dot plots of Downregulated and Upregulated AML miRNA signature in a second cohort of AML patients at diagnosis compared to HD (**B**) ROC curves reflecting the performance of the individual features of the 9 miRNA signature in discriminating AML patients from healthy donors. (**C**) ROC plot showing the discriminatory value of the 9-miRNA signature in the second cohort. AUROC: Area Under ROC; CI: 95% Confidence Intervals; *p*-value: correspond to a Mann-Whitney U test addresses testing the null hypothesis of the AUROC is 0.5 (i.e., the classifier is random).

This miRNAs signature was also confirmed in the second cohort (Figure 3A–C).

### 2.2. Functional Analysis of the Pathways of AML Bone Marrow Circulating miRNAs

GO classification and KEGG pathway analyses were used to identify the biological functions of miRNA pathways, including oncogenic processes, cell death pathways, immune functions and proliferation signaling pathways. The threshold of GO and KEGG classification was set as *p* < 0.05. MicroRNAs in cancer (hsa:05206, *p* = 6.05 × 10^−11^), acute myeloid leukemia apoptosis (hsa:05221, *p* = 3.98 × 10^−5^), Ras signaling pathway (hsa:05221, *p* = 2.29 × 10^−5^) and PI3K-Akt signaling pathway (hsa:04151, *p* = 1.82×10^−4^) were the most significantly enriched miRNA target gene pathways. We also observed miRNAs implicated in apoptosis, chemokine and T cell receptor signaling pathways (Table 3A). GO classification analysis of DE miRNAs also showed several biological processes, cellular components, and molecular functions implicated in the regulation of macromolecule metabolic processes, positive cell proliferation, negative regulation of cell death, regulation of apoptosis and immune processes (Table 3B). This suggests the implication of these deregulated circulating miRNAs both in malignant cell proliferation and the immune tumor microenvironment of AML.

The analysis of signaling pathways related to the published data (see the discussion section) showed the involvement of several of these confirmed DE miRNAs, such as miR-21, miR-24 and miR-9, in T cell activation, proliferation and Th2 and Treg polarization. Some of these DE miRNAs are considered oncogenes and are expressed in AML tumor cells, suggesting their role in tumor protection and T cell dysfunction.

### 2.3. Prognosis Value of Bone Marrow Circulating miRNAs in AML Based on Genetic Risk.

We compared the miRNA profiles of newly diagnosed patients based on their genetic risk classification to highlight the correlation between circulating bone marrow miRNAs and the prognostic value of classical genetic risk. Given the experimental limits and the limited number of patients, we compared two groups of patients, those considered favorable (FAV) (*n* = 9) and those considered adverse (ADV) (*n* = 10), according to their prognosis of molecular and genetic risk. The list of miRNAs was filtered with a *p*-value < 0.05 and fold change ≥ 1.5. Our analysis identified 19 downregulated miRNAs in the favorable group compared to the adverse group, and only miRNA-222 was upregulated (FC = 30; *p* = 0.04) (Table 4). Interestingly, six of these downregulated miRNAs in the FAV group were also downregulated in all AML patients at diagnosis compared to HD. However, miR-196b and 490-3p were downregulated in both the FAV group and the HD group compared to all AML patients (Table 4). This suggests a link between some DE BM circulating miRNAs and classical genetic risk classification.

### 2.4. Correlation between Bone Marrow Circulating miRNA Expression Profiles and Overall Survival Rate of Patients with AML

**Figure 4:**
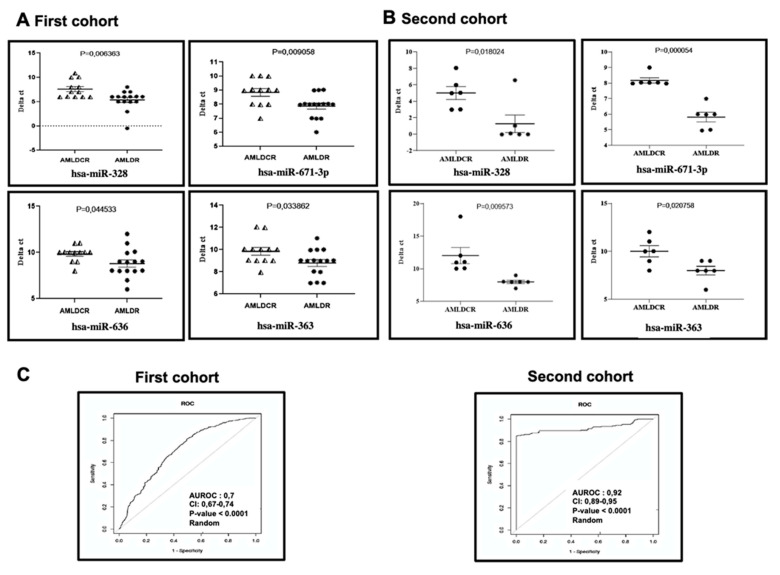
Prognosis value of AML bone marrow circulating miRNAs. The expression dot blot and receiver operating characteristic (ROC) curves reflecting the representative bone marrow circulating miRNAs (4 miRNAs) differentially expressed between the AML patients based on their overall survival at diagnosis (**A**) and validated in an independent second cohort of patients (**B**). (**C**) Receiver Operating Characteristic (ROC) plot validating the combined prognosis power of miR-671-3p and miR-328 in the first and second cohorts. AMLDCR: AML at diagnosis samples in which the patients have complete remission 4 years post diagnosis. AMLDR: AML at diagnosis samples in which the patients relapsed and died during the 4 years post diagnosis.

Regarding cytogenetic risk, the AML circulating bone marrow miRNA profile revealed a set of differentially expressed miRNAs between the unfavorable and favorable groups. Regardless of the classic classification factors, we compared patients only on the basis of their overall survival 4 years after diagnosis. Comparison of the two groups of patients at the time of diagnosis showed that 4 miRNAs (miR-328; miR-671-3p; miR-636 and miR-363) were downregulated in patients who remained in remission 4 years after treatment (*n* = 12) compared to those who died during the first three years after treatment (*n* = 15) (Figure 4A). We confirmed these downregulated miRNAs in a small second cohort (12 AML patients at diagnosis: 6 AMLDCR vs 6 AMLDR) (Figure 4B). These miRNAs could be used as independent prognostic factors for overall survival. In particular, the prognostic relevance of miR-328 and miR-671-3p expression was more statistically significant in separating the patient group based on their OS than based on their genetic risk, and the combined ROC analyses revealed an AUC of 0.7 in first and 0.92 in second cohort (*p* < 0.0001), indicating a prognostic value of these 2 miRNAs (Figure 4C). In conclusion, our analysis showed that the expression of circulating bone marrow miRNAs appears to be associated with overall survival risk.

### 2.5. Bone Marrow Circulating miRNAs Profile of First Complete Remission AML Patients (AMLPT) 

To understand the importance of these DE miRNAs described previously in defining the response to treatment, we analyzed and compared the miRNA profiles of twelve paired patients at diagnosis to their profiles during first complete remission (CR1) three months post treatment (PT). Our results showed that 19 miRNAs were upregulated and 69 were downregulated in CR1 AML patients after treatment (AMLPT) compared to newly diagnosed patients (AMLD) (*p* < 0.05 and 1,5-fold change) (Table 5). 

To determine the prognostic value of these miRNAs in determining posttreatment remission, we compared the profiles of miRNAs differentiating AML patients in CR1 (AMLPT) and healthy donors (HD) from those of newly diagnosed patients (AMLD). The results showed that 14 miRNAs are upregulated, and four miRNAs were downregulated in AMLD compared to both AMLPT patients and healthy donors. 

**Figure 5:**
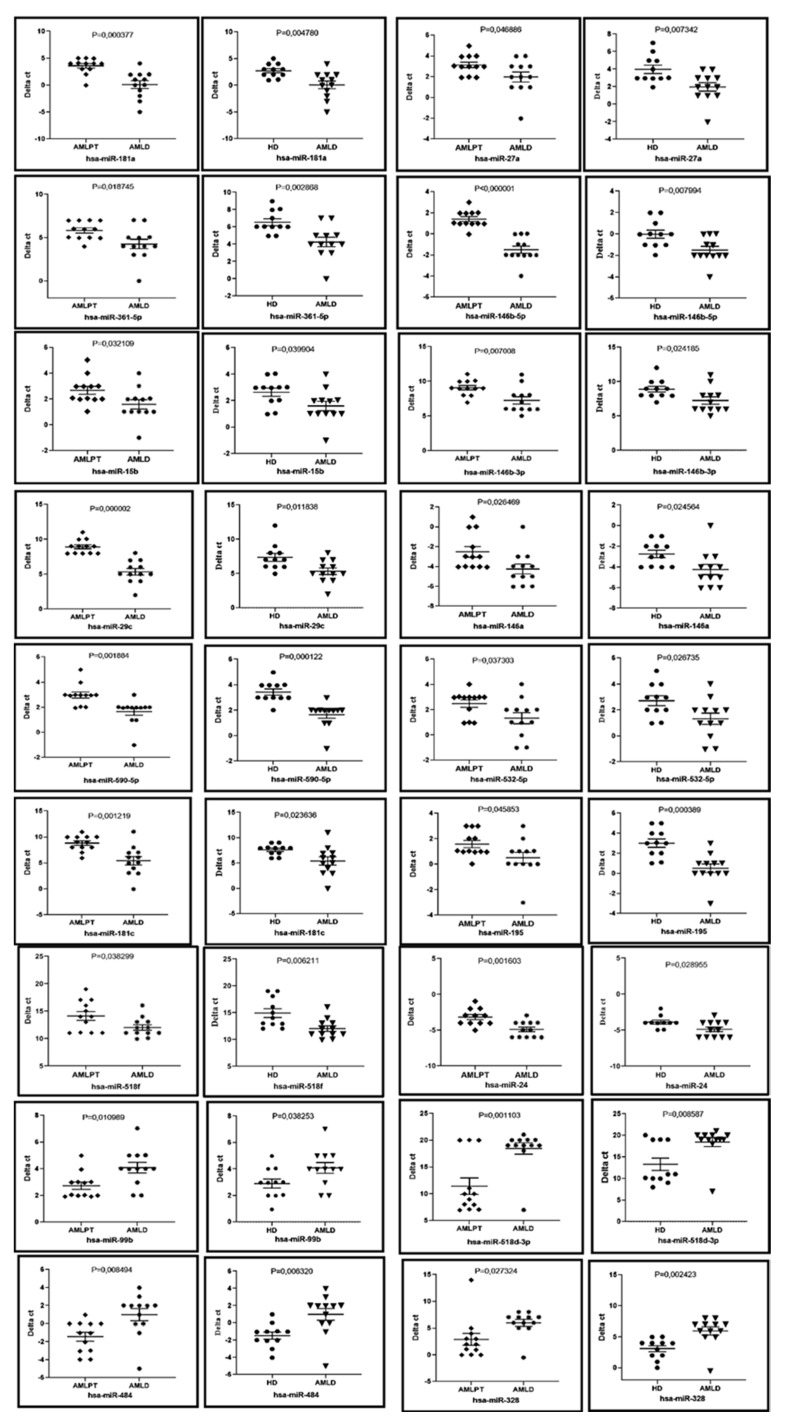
Prognosis value of AML bone marrow circulating miRNAs related to remission after treatment. Correlated differentially expressed miRNAs between AMLD vs. AMLPT patients and AMLD patients vs. HD. The expression dot plot curves reflecting the correlated differentially expressed bone marrow circulating miRNAs in AML patients at diagnosis (*n* = 12) compared to both healthy donors (*n* = 11) and the same patients in relative remission (blast < 5%) post treatment (*n* = 12). AMLD: AML patients at diagnosis. HD: Healthy donors. AMLPT: AML first complete remission post treatment patients.

Interestingly, the results showed that the bone marrow expression level of miR-518d-3p was also still undetectable in CR1 patients after treatment similar to HD (Figure 5). Overall, this correlation allowed us to establish promising bone marrow circulating miRNA biomarkers for AML remission post treatment, suggesting their possible role in the response of AML patients to chemotherapy treatment.

## 3. Discussion

Despite advances in the search for new optimal therapeutic approaches, including targeted immunotherapy, only a minority of patients are cured, especially in the elderly population. The identification of new therapeutic targets is necessary to design new or improve existing therapeutic protocols. This is only possible through understanding the different players that drive the pathogenesis of this disease, especially those related to TME. In addition, the search for new diagnostic and prognostic markers of AML is necessary, given the heterogeneity of the disease and the inadequacy of current genetic markers in predicting survival. MiRNAs circulating free or contained in microvesicles (MVs) can be secreted by a wide variety of cells in the TME and are increasingly seen as potential future markers for the diagnosis and prognosis of various malignant diseases.

In this work, we have for the first time analyzed the profile of enriched miRNAs circulating in the bone marrow, which is considered the main location of the initiation and development of acute myeloid leukemia. The results demonstrated a consistent number of differentially expressed miRNAs between AML patients and healthy donors. This allowed us to establish a miRNA signature at the time of diagnosis that provided good separation between the patients and healthy donors. More than thirty of these miRNAs were validated in a second independent cohort of patients. However, two DE miRNAs in the first cohort did not give similar results in the second cohort, of which 14 were similarly DE but statistically insignificant. This could be due to the limited number of patients in the second cohort, which suggests the need to increase the number of patients in the future. Interestingly, some of these DE miRNAs were undetectable or expressed at very low levels either in patients or in HD, suggesting their role in the development of AML. Among the miRNAs that were expressed in HD but undetectable in patients, we found miR-448, miR-890 and miR-504. These miRNAs have been shown to be downregulated and function as tumor suppressors in several types of cancer [24,25]. Recently, miR-504 was shown to be significantly downregulated in the serum of AML patients and cell lines, and its overexpression inhibits cell growth and induces apoptosis in leukemic cells [26]. MiR-448 is downregulated in T-cell acute lymphoblastic leukemia (T-ALL) and seems to target the principal proto-oncogene (TAL1) of this disease [27]. To date, no publication has shown a link between miR-448 and miR-890 in AML, suggesting that their deregulation in bone marrow fluid may be associated with cells other than leukemic blasts. Their deregulation in the TME following the development of the disease must be elucidated for their direct impact not only on the proliferation of malignant cells but also on immune TME cells. Interestingly, it was reported that in human colon cancer, miR-448 could suppress CD8+ T-cell apoptosis and enhance the CD8+ T-cell response by inhibiting indoleamine 2,3-dioxygenase 1 enzyme function [28]. In our results, we also observed other differentially expressed miRNAs, including miR-520a, 651, 520f and 449, that were upregulated in patients and undetectable or expressed at very low levels in HD. Among these, only miR-520a was described to be DE in AML blasts and was associated with a poor prognosis in AML [29], and miR-520f has been reported as an oncogene in human melanoma and promotes malignant cell proliferation [30].

Our results also allowed us to detect miRNAs that are strongly DE but are expressed in both the AML and HD groups. Some, such as miR-9, miR-548d and miR-21, are highly deregulated and are known to be oncogenes in AML or other types of cancer. An increased level of miR-9 has been observed in the bone marrow and peripheral blood of 200 AML patients compared to HD [31]. Chen Tian et al. showed that the knockdown of miR-9 suppressed the proliferation of AML cells by the induction of G0 arrest and apoptosis in vitro, resulting in decreased circulating leukemic cells and prolonged survival in a xenotransplant mouse model [32]. In addition, overexpression of miR-9 enhances the suppressive function of MDSCs, while its knockdown impairs them and inhibits the tumor growth of Lewis lung carcinoma in mice. [33]. MiRNA-548d-5p was shown to be upregulated in metastatic colorectal cancer tumor-derived exosomes [34]. There was no association between these miRNAs and AML in the literature, suggesting that upregulation of AML in bone marrow fluid is due to a cell origin other than leukemic cells. Certain studies have shown its role in promoting osteogenic differentiation of mesenchymal stem cells [35,36]. Surprisingly, we also observed that some AML-upregulated bone marrow circulating miRNAs are reported in the literature as tumor suppressors, including miR-34a, miR-375, miR-576-3p, and miR-597. For example, miR-34a is strongly expressed in AML patient bone marrow fluid and is known to be downregulated in leukemia cells in the marrow and peripheral blood serum of patients [37,38] and appears to play an important role as a tumor suppressor and immunotherapeutic agent [39,40]. The expression of miR-375 was also downregulated in leukemic cell lines and primary AML blasts compared to normal controls, and its overexpression decreased proliferation and colony formation, reduced xenograft tumor size and prolonged the survival time in a leukemia xenograft mouse model [41]. MiR-576-3p was shown to be downregulated in colorectal [42] and bladder cancer [43], to inhibit the migration and invasion of lung adenocarcinoma [44] and to significantly inhibit the migration and pro-angiogenic abilities of glioma cells under hypoxic conditions [45]. Additionally, miR-597 inhibits breast and colon cancer cell proliferation, migration and invasion [46,47]. These findings strengthen our previous hypothesis for a cell origin other than leukemia cells for these miRNAs and suggest their dual function according to the type of cancer or the tumor focus. Interestingly, recent analyses of the regulatory network of CD19-CAR-T immunotherapy for B acute lymphocyte leukemia revealed that microRNA miR-375 could regulate the crosstalk between the genes encoding transcription factors and histones involved in CD19-CAR-T therapy [48].

Our results also showed that some miRNAs were expressed in both the AML and HD groups but were significantly downregulated in AML patients compared to HD, among which we found miR-145, 150, 204, 365 and 518b. MiR-145 has been increasingly identified as a critical suppressor of carcinogenesis and therapeutic resistance in several cancers [49]. Recent data showed that the level of this miRNA in serum and bone marrow mononuclear cells of AML patients was significantly lower than that of HD and was related to poor prognosis [50]. MiR-145 seems to play an important role in the immune response against leukemic cells. It was reported to enhance host antitumor immunity by altering the cytokine milieu, metastatic microenvironment and reprogramming tumor-associated myeloid cells [51]. Recent work revealed that miR-145, which is known to be downregulated by cisplatin in cisplatin-resistant ovarian cancer cells, also represses PD-L1 gene expression and induces T cell apoptosis in vitro [52]. It is known that the level of miR-150 is decreased in the plasma and blast cells of AML patients at diagnosis and that reintroducing miR-150 expression induces myeloid differentiation and inhibits the proliferation of AML cells [18,53]. Xi et al. developed a novel targeted therapeutic strategy using FLT3L-guided miR-150-based nanoparticles to treat FLT3-overexpressing AML in an animal model with high efficacy and minimal side effects [54]. MiR-204 expression in AML patients was decreased and was associated with shorter patient survival. Higher expression of this miR was observed in patients after induction therapy and was correlated with complete remission [55], and its targeting promotes the viability and invasion of AML cells [56]. Wang et al. demonstrated that overexpression of these miRNAs potentiates the sensitivity of AML cells to arsenic trioxide [57]. In addition, recent work showed that in breast cancer, miR-204 regulated the expression of key cytokines in tumor cells and reprogrammed immune cells by shifting myeloid and lymphocyte populations, suggesting that this miRNA suppresses tumor metastasis and remodeling the immune microenvironment [58]. MiR-518b was also reported to function as a tumor suppressor in glioblastoma [56], esophageal and squamous cell carcinoma [59].

Based on these results, we also established an AML signature at diagnosis, consisting of nine miRNAs with an AUC greater than 0.77. Combined ROC analyses using these nine miRNAs revealed an elevated AUC of 0.98 (*p* < 0.0001), indicating an additive effect on the diagnostic value of these miRNAs. This signature, as well as more than thirty DE miRNAs, was validated in a second cohort of patients and provided good separation between AML patients and HD. Our patient group comparison at diagnosis based on their overall survival showed that miR-328, miR-671-3p, miR-636 and miR-363 are upregulated in relapsed patients. These miRs are described in the literature as promising prognostic biomarkers in AML or other malignant diseases [21,60,61,62,63,64,65,66,67,68,69,70,71,72,73,74,75]. Interestingly, all the patients involved in this comparison were not grafted, which suggests that these four miRs could constitute a signature to guide the choice of the best treatment strategy, especially the utility of grafting patients at diagnosis. A recent study using a TCGA dataset identified a set of miRNAs, including miR-363, that could predict clinical outcome in a heterogeneous AML population [76]. This study confirms our results showing the high expression of circulating bone marrow miR-363 in nontransplanted patients with shorter OS and suggests that its high expression may help to identify patients recommended for an early allo-HSCT regimen. Interestingly, this miRNA seems to play an important role in the immune response against leukemic cells. An enrichment of specific cellular miRNAs, including miR-363, in EVs derived from CD40/IL-4-stimulated chronic lymphoblastic leukemia cells was observed, and autologous patient CD4(+) T cells were found to be capable of internalizing the CLL-EVs that target the immunomodulatory molecule CD69 [77].

Our comparison of patients before and after three months of treatment (first complete remission (CR1)) allowed us to identify a set of miRNAs that were differentially expressed (4 downregulated and 14 upregulated) in the same way as when comparing patients at diagnosis with HD. However, other analyses are underway on a larger cohort of patients to study the long-term prognostic value (four years after treatment) of these miRNAs. It should also be noted that an analysis of the profile of relapsing patients during the first months is also necessary to validate these miRNAs, which we could not obtain in our study. These miRNAs, in addition to their value in monitoring posttreatment remission, may also play a role in the elimination of blasts directly or indirectly by improving the antileukemia response. Among these DE in AML CR1, miR-484, miR-518d and miR-99b are upregulated in addition to the previously described miR-328. MiR-99b was also showed as being responsible for the conversion of monocytes into MDSCs and to be associated with resistance to treatment with immune checkpoint inhibitors in melanoma patients [78]. MiR-518d may be an interesting target due to its low or undetectable level in HD and post treatment. It was reported to be implicated in the inhibition of cervical cancer cell proliferation, migration and invasion [79] and in the regulation of advanced small cell lung cancer cell proliferation, migration and chemotherapy response [80]. To date, little work has focused on this miRNA, and none of the studies have reported its involvement in AML, which makes it a good avenue for future investigation. For the downregulated miRNAs in AML CR1, miR-24 appear to be involved in AML evolution an immune response. High expression of miR-24 has been observed in AML leukemia patients and is associated with the risk of relapse and poor survival [81], and its role in nasopharyngeal carcinoma pathogenesis by mediating T-cell suppression has been demonstrated [82]. In a previous study, we demonstrated that this miRNA negatively regulates IFN-γ expression in T lymphocytes [83], and recently, we showed its direct impact on AML T lymphocyte fragility and dysfunction (in preparation). Furthermore, other downregulated miRNAs in CR1, such as miR-15b [84], miR-27a [85], miR-29c [86], miR-106a [87] and miR-181a [88], have been described as being implicated in AML pathogenesis or drug resistance.

As reported, high expression of miR-181a in AML blasts, cell lines and serum has been observed [89,90], and its overexpression significantly enhanced cell proliferation and increased the ratio of S-phase cells by regulating the tumor suppressor ATM [91,92]. Inhibition of this miR in mice implanted with AML CD34+ hematopoietic progenitor cells (HSPCs) increased myeloid differentiation, inhibited engraftment and infiltration of leukemic CD34+ cells into the bone marrow and spleen, and finally reduced leukemic symptoms [93]. However, miR-181a also plays a role in the sensitization of leukemic resistant cells to daunorubicin and NK cells and may provide a promising option for AML immunotherapy treatment of chemoresistant blasts [94]. MiR-181a appears to play an important role in T cell activation [95] and CAR-T cell immunotherapy [96]. A recent study showed that inhibition of DOT1L reduces the expression of miR-181a, which in turn selectively enhances low-avidity T cell responses and attenuates graft versus host development, suggesting that this would allow the safe and effective use of allogeneic antitumor T cells [97]. In melanoma patients, the plasma levels of miR-181a were higher in patients at diagnosis compared to controls, and the development of metastasis has been associated with changes in immune effector and regulatory cells with changes in plasma and cellular levels of immune regulatory miRNA [98]. Melanoma-derived exosomes decrease TNFα secretion in CD8+ cells and are enriched for hsa-miR-181a, which is capable of interacting directly with the 3′-UTR sequence of TNFα and driving immune escape in melanoma [99]. We have previously shown in our laboratory that overexpression of miR-181a negatively regulates IFN-γ expression in activated PB CD4+ T cells by directly binding to its target sites in the mRNA and increases the expression of IL4 [83]. MiR-181c also seems to play a dual role similar to miR-181a in both malignant and immune cells. A study conducted by R Su et al. compared patients with AML blasts with normal controls, and most AML patients showed significantly increased expression levels of all miR-181 members, including miR-181c [100]. MiR-181c is important in T cell activation, and the overexpression of miR-181c results in inhibition of T cell activation and actin polymerization coupled with defective immunological synapse formation [101]. Interestingly, R. Le Dieu et al. showed that the ability of AML T cells to form immune synapses was significantly impaired [102]. It was identified as a novel miRNA that promotes Th17 cell differentiation and autoimmunity [103]. In addition, circulating miR-181c can be of a cell origin other than cancer cells. Xiao Li et al. showed that exosomes derived from human umbilical cord mesenchymal stem cells are enriched for miR-181c, which in turn attenuates burn-induced excessive inflammation [104].

MiR-532 was reported as an oncogene in breast [105], colorectal [106] and human gastric cancers [107]. In recent published work, Lin et al. concluded that plasma exosome-derived miR-532 can be used as a novel survival predictor for acute myeloid leukemia [108]. This miRNA has also been implicated in the immune inflammatory response [109]. In addition to its function as a tumor suppressor and/or oncogene [110], miR-146a was shown to play a critical role in regulating T cell functions by providing a general brake on proliferation and activation of immune cells and was able to limit immune responses linked to the TH1 and TH17 subsets. Mice lacking this miRNA developed more severe TH17 responses in murine models of autoimmunity, with increased production of IFN-γ and IL-17 and reduced secretion of IL-4 [111]. Furthermore, miR-146a is also a key regulator of Treg cell biology, as deficiency of miR-146a leads to impaired function of Treg cells and consequently to the breakdown of immunological tolerance and to the development of fatal immune-mediated lesions depending on IFN-γ. Indeed, in addition to their role in the proliferation of cancer cells, many of these downregulated miRNAs, including miR-15 [112,113], miR-27a [114,115] and miR-29c [116,117,118], appear to play a role in immune regulation, T regulatory and inflammatory responses and TIL dysfunction. 

Together, these results suggest that enriched circulating bone marrow miRNAs are key regulators of AML blast proliferation and affect immune cell functions in the TME. Our GO classification and KEGG pathway analyses confirmed that these DE circulating miRNAs could play a role not only in the oncogenic process, cell death and proliferation signaling pathways but also in immune function. Further research may elucidate their role in the malignant cells and TME. Studies are currently underway in our laboratory to investigate the impact of these miRNAs on the antileukemic immune response with the aim of establishing new immunotherapeutic targets of AML.

## 4. Materials and Methods

### 4.1. Patients

Patients used in this study had a newly diagnosed AML in addition to being in complete remission after the first treatment (three months after first induction) as determined by bone marrow test. A total of 46 patients at the time of diagnosis, 12 of them in complete remission post treatment, provided bone marrow samples. Healthy subjects (*n* =11) were enrolled as negative controls. None of these controls had been previously diagnosed with any type of malignancy or other benign disease. Informed consent, approved by the Clinical Research Ethics Committee of Jules Bordet Institute (approval code: B079201214544, approval date: 5 July 2012) was obtained from each participant. Details of the clinical data are provided in Table 6. 

### 4.2. Bone Marrow Aspiration Sampling and RNA Extraction.

At presentation, bone marrow aspiration samples for miRNA detection were collected in EDTA-K2 tubes and processed within 1 h of collection. Bone marrow body fluid samples were centrifuged at 1200× *g* for 10 min at 4 °C to pellet the hematopoietic cells, and the supernatant was transferred into microcentrifuge tubes, followed by a second centrifugation at 12,000× *g* for 10 min at 4 °C. The supernatant was transferred to RNase/DNase-free tubes and stored at −80 °C. Total RNA was isolated from the plasma using a mirVana PARIS isolation kit (Ambion, Austin, TX, USA) according to the manufacturer’s instructions for plasma samples. Briefly, 400 μL of human bone marrow plasma was used to extract total RNA. Each sample was eluted in 100 μL of RNase-free water and concentrated to a final volume of 20 μL by using an Eppendorf Concentrator Plus 5301 (Eppendorf, Aarschot, Belgium). The RNA sample concentration was quantified by a NanoDrop ND-1000 (Thermo Fisher Scientific, Waltham, MA, USA).

### 4.3. miRNA Expression Profile

Thirty nanograms of total bone marrow fluid extracted RNA was used for cDNA synthesis from the miRNAs using a TaqMan^®^ microRNA Reverse Transcription Kit (#4366596; Applied Biosystems, Waltham, MA, USA) and Megaplex RT primers (Human Pool A, #4399966; Applied Biosystems) following the manufacturer’s protocol described previously [18]. After the preamplification step, the products were amplified and then loaded into TaqMan Human MicroRNA Array A (#4398965; Applied Biosystems), which contains the TaqMan primers and probes in each well for the 380 different mature human miRNAs; miR-425 transcript was used as a normalization signal. The relative expression levels of miRNAs were calculated using the comparative ΔΔ*C*t method as described previously [18]. The fold changes in miRNAs were calculated by the equation 2^−ΔΔ*C*t^.

### 4.4. TaqMan miRNA Assay for Individual miRNAs

The expression of individual miRs was determined using the TaqMan miRNA assay as described previously [18]. The expression levels of miRNAs were calculated as described previously, and miR-425 was used as an internal reference. The predesigned TaqMan assay primers and probes corresponding to the ones used in TLDA card A were purchased from Thermo Fisher Scientific. The calculated delta Ct ± SD for the patients was compared with the delta Ct ± SD (SD stands for the standard deviation of the average delta *C*t of the group for the healthy control group and tested for statistical significance).

### 4.5. Statistical Analysis

Data analysis was performed in R version 3.5.3 using packages of the Bioconductor project (DOI: 10.1186/gb-2004-5-10-r80). Raw cycle threshold (*C*t) values were loaded into R using the HTqPRC package (doi:10.1093/bioinformatics/btp578) and flagged as “Undetermined” if they were above 35. miRNAs that are expressed in at least one sample group (i.e., *C*t > 35 in at least X samples) were retained for further analysis. To normalize the data, delta *C*t (Δ*C*t) values were calculated for each sample by subtracting the *C*t value of miR-425 from its other values. miR-425 was selected as the best reference candidate by both the geNorm and NormFinder methods implemented in the NormqPCR package (https://rdrr.io/bioc/NormqPCR/man/NormqPCR-package.html) accessed on 1 August 2020. Testing for differential expression between sample groups was performed using empirical Bayes moderated t-statistics computed by the limma package (https://bioconductor.org/packages/release/bioc/html/limma.html). miRNAs with an absolute fold change > 1.5 and a *p* value < 0.05 were considered differentially expressed (DEmiRNAs). The ability of the selected signatures and miRNAs to accurately discriminate between different patient groups was estimated by constructing and assessing the performance of ensemble-based classifiers (DOI 10.1007/s10462-009-9124-7). These are stacked classifiers that combine the predictions of several classification algorithms in order to predict the outcome. The Area Under Receiver Operating Characteristic Curve (AUC) was calculated by a bootstrapping procedure with 100 iterations. Enrichment analysis of Gene Ontology (GO) terms and Kyoto Encyclopedia of Genes and Genomes (KEGG) pathways (https://www.genome.jp/kegg/pathway.html) was performed using the Database for Annotation, Visualization, and Integrated Discovery (DAVID) tool (http://david.abcc.ncifcrf.gov/) accessed on 13 August 2020. 

## 5. Conclusions

In our study, for the first time, we highlight circulating miRNAs in the bone marrow of AML patients and provide important insight into the possible role of these miRNAs in the TME. We have shown that the main TME of AML is well enriched in molecules that have variable expression according to the progression of the disease as well as the predisposition to the treatment response. In addition to the value that these circulating miRNAs can provide for the diagnosis of AML or its confirmation, their contribution could extend to the prognostic level by guiding, at diagnosis, the choice of transplants for certain patients. Some of these miRNAs appear to be of malignant origin, and their impact on resistance to treatment or evasion of immune system surveillance has been described in other types of cancer. Other miRNAs that to date have never been described in AML could have another cellular origin (immune cells, MSCS and stromal cells) and could be at the origin of the promotion of the proliferation of blasts while directing the immune response to a protumoral phenotype. Further in vitro and in vivo investigations are necessary to confirm these hypotheses, which open a wide door to new immunotherapeutic targets for the eradication of AML.

## Figures and Tables

**Table 1:** Bone marrow circulating miRNAs differentially expressed in AML newly diagnosed compared to healthy donors.

**Up regulated miRNAs in AMLD vs. HD**		
**MiR Connotation**	***p*** **Value**	**FC AMLD1 vs. HD**
hsa-miR-520a-3p	0.0000004	103.59
hsa-miR-548b-5p	0.0000187	15.35
hsa-miR-651	0.0007066	13.01
hsa-miR-449b	0.0029965	7.84
hsa-miR-520f	0.0012403	7.19
hsa-miR-330-5p	0.0476803	6.55
hsa-miR-34c-5p	0.0300460	5.10
hsa-miR-9	0.0030388	15.45
hsa-miR-34a	0.0008391	14.22
hsa-miR-548d-5p	0.0000390	13.09
hsa-miR-655	0.0061917	8.55
hsa-miR-548c-5p	0.0002937	7.99
hsa-miR-135a	0.0225966	6.95
hsa-miR-502-5p	0.0028352	6.68
hsa-miR-576-3p	0.0008681	5.99
hsa-miR-449a	0.0003304	5.97
hsa-miR-518f	0.0015237	5.88
hsa-miR-21	0.0000118	5.61
hsa-miR-629	0.0000000	5.60
hsa-miR-195	0.0000050	5.32
hsa-miR-517c	0.0011033	5.02
hsa-miR-597	0.0026663	4.94
hsa-miR-199b-5p	0.0421635	4.85
hsa-miR-548d-3p	0.0203888	4.76
hsa-miR-181a	0.0039392	4.76
hsa-miR-570	0.0180477	4.75
hsa-miR-660	0.0000101	4.56
hsa-miR-202	0.0100613	4.24
hsa-miR-24	0.0225184	4.24
hsa-miR-375	0.0016871	4.18
hsa-miR-130b	0.0000115	3.81
hsa-miR-361-5p	0.0020377	3.65
hsa-miR-451	0.0019980	3.58
hsa-miR-181c	0.0131902	3.49
hsa-miR-579	0.0002417	3.49
hsa-miR-362-3p	0.0106066	3.22
hsa-miR-25	0.0005764	3.15
hsa-miR-29c	0.0095014	3.00
hsa-miR-511	0.0103669	2.90
hsa-miR-146b-3p	0.0096730	2.88
hsa-miR-532-5p	0.0014276	2.70
hsa-miR-106a	0.0012818	2.62
hsa-miR-874	0.0089434	2.61
hsa-miR-212	0.0012351	2.50
hsa-miR-598	0.0046231	2.48
hsa-miR-221	0.0144840	2.47
hsa-miR-18a	0.0052162	2.46
hsa-miR-27a	0.0160879	2.45
hsa-miR-590-5p	0.0015733	2.40
hsa-miR-20b	0.0471303	2.36
hsa-miR-101	0.0162702	2.23
hsa-miR-146a	0.0174218	2.22
hsa-miR-142-3p	0.0246946	2.21
hsa-miR-146b-5p	0.0305051	2.14
hsa-miR-93	0.0175670	1.97
hsa-miR-140-5p	0.0362266	1.96
hsa-miR-18b	0.0224577	1.90
hsa-miR-423-5p	0.0088564	1.87
hsa-miR-92a	0.0443479	1.71
hsa-miR-210	0.0429423	1.68
hsa-miR-483-5p	0.0423541	1.62
**Downregulated miRNAs in AMLD vs. HD**		
**MiR Connotation**	***p* Value**	**FC AMLD1 vs. HD**
hsa-miR-326	0.00663326	0.103
hsa-miR-198	0.00282623	0.078
hsa-miR-518d-3p	0.00001285	0.018
hsa-miR-107	0.00000130	0.012
hsa-miR-215	0.00082673	0.009
hsa-miR-504	0.00000004	0.006
hsa-miR-890	0.00000995	0.001
hsa-miR-448	0.00007498	0.001
hsa-miR-532-3p	0.05245365	0.616
hsa-miR-342-3p	0.04884326	0.602
hsa-miR-200c	0.04994680	0.547
hsa-miR-491-5p	0.01187570	0.543
hsa-miR-28-3p	0.00718598	0.531
hsa-miR-99b	0.00686813	0.444
hsa-miR-574-3p	0.01232231	0.432
hsa-miR-744	0.00082444	0.358
hsa-miR-190	0.05420973	0.353
hsa-miR-150	0.00317263	0.322
hsa-miR-197	0.00005892	0.270
hsa-miR-125a-5p	0.00614751	0.262
hsa-miR-203	0.02014882	0.258
hsa-miR-891a	0.02627127	0.248
hsa-miR-485-3p	0.05481495	0.247
hsa-miR-489	0.00938728	0.245
hsa-miR-204	0.00027158	0.207
hsa-miR-193b	0.00487572	0.203
hsa-miR-184	0.01694239	0.203
hsa-miR-484	0.00012474	0.173
hsa-miR-618	0.03435969	0.152
hsa-miR-134	0.01390311	0.145
hsa-miR-433	0.04689247	0.135
hsa-miR-145	0.00000042	0.120
hsa-miR-149	0.02704296	0.119
hsa-miR-328	0.00004261	0.105
hsa-miR-654-5p	0.03086797	0.079
hsa-miR-365	0.00000686	0.066
hsa-miR-518b	0.00169071	0.063

**Table 2:** Confirmed differentially expressed microRNAs between AML patients and HD in a second cohort.

MiRNAs Connotation	*p* Value AMLD1	FC AMLD1	*p* Value AMLD2	FC AMLD2	*p* Value AMLD Total	FC. AMLD Total
hsa-miR-520a-3p	4.08 × 10^−7^	103.58	0.016	3.724	9.22 × 10^−5^	31.587
hsa-miR-9	0.003	15.451	3.20 × 10^−5^	41.678	0.0002	22.022
hsa-miR-548b-5p	1.87 × 10^−5^	15.354	0.22	2.003	0.0026	7.418
hsa-miR-34a	0.0008	14.221	0.006	13.443	6.19 × 10^−5^	13.938
hsa-miR-548d-5p	3.89 × 10^−5^	13.091	0.021	5.268	7.85 × 10^−5^	9.458
hsa-miR-651	0.0007	13.011	0.004	7.129	0.0006	10.496
hsa-miR-548c-5p	0.0002	7.993	0.150	1.961	0.0031	4.839
hsa-miR-449b	0.0029	7.843	0.007	6.301	0.0019	7.253
hsa-miR-520f	0.0012	7.190	0.001	3.562	0.0011	5.594
hsa-miR-502-5p	0.0028	6.676	0.132	2.898	0.0109	4.955
hsa-miR-576-3p	0.0008	5.989	2.11 × 10^−5^	8.364	2.68 × 10^−5^	6.748
hsa-miR-449a	0.0003	5.972	0.078	2.814	0.0021	4.565
hsa-miR-21	1.17 × 10^−5^	5.611	1.76 × 10^−7^	8.913	8.07 × 10^−8^	6.619
hsa-miR-629	2.98 × 10^−8^	5.600	2.23 × 10^−7^	3.693	3.41 × 10^−9^	4.826
hsa-miR-195	5.006 × 10^−6^	5.315	0.20	1.546	0.002	3.419
hsa-miR-517c	0.0011	5.019	0.35	1.264	0.020	3.068
hsa-miR-597	0.0026	4.941	0.0008	8.215	0.0001	5.925
hsa-miR-181a	0.0039	4.760	0.0161	2.755	0.0044	3.916
hsa-miR-660	1.00 × 10^−5^	4.558	0.0590	1.536	0.0003	3.091
hsa-miR-375	0.001	4.177	0.0009	9.544	0.0001	5.611
hsa-miR-130b	1.14 × 10^−5^	3.815	0.1794	1.766	0.0077	2.897
hsa-miR-361-5p	0.002	3.651	0.0168	1.920	0.0025	2.902
hsa-miR-451	0.0019	3.584	0.0128	2.659	0.0016	3.221
hsa-miR-579	0.0002	3.487	0.0290	1.927	0.0006	2.821
hsa-miR-25	0.0005	3.154	5.15 × 10^−5^	4.060	2.71 × 10^−5^	3.451
hsa-miR-106a	0.0012	2.616	0.07	1.558	0.0050	2.174
hsa-miR-212	0.0012	2.501	0.49	1.003	0.0251	1.805
hsa-miR-598	0.0046	2.482	0.24	1.346	0.0280	1.994
hsa-miR-18a	0.0052	2.459	0.0008	3.101	0.0011	2.671
hsa-miR-744	0.0008	0.358	3.03 × 10^−5^	0.201	8.80 × 10^−5^	0.291
hsa-miR-150	0.0031	0.322	1.10 × 10^−5^	0.116	0.0001	0.223
hsa-miR-197	5.8910^−5^	0.270	0.19	0.734	0.0041	0.386
hsa-miR-204	0.0002	0.207	1.39 × 10^−5^	0.113	8.73 × 10^−6^	0.167
hsa-miR-193b	0.0048	0.203	9.67 × 10^−7^	0.052	0.0001	0.125
hsa-miR-484	0.0001	0.173	0.11	0.552	0.0023	0.262
hsa-miR-145	4.21 × 10^−7^	0.120	4.47 × 10^−6^	0.112	3.11 × 10^−9^	0.117
hsa-miR-328	4.26 × 10^−5^	0.105	0.42	1.135	0.014	0.245
hsa-miR-198	0.0028	0.078	0.22	0.409	0.013	0.141
hsa-miR-365	6.86 × 10^−6^	0.066	0.0005	0.132	3.58 × 10^−6^	0.084
hsa-miR-518b	0.001	0.063	4.28 × 10^−5^	0.007	5.05 × 10^−5^	0.028
hsa-miR-518d-3p	1.28 × 10^−5^	0.018	0.37	0.656	0.005	0.066
hsa-miR-107	1.30 × 10^−6^	0.012	0.11	0.174	0.0004	0.031
hsa-miR-215	0.0008	0.009	0.06	0.083	0.0024	0.019
hsa-miR-504	3.50 × 10^−8^	0.006	5.70 × 10^−6^	0.012	1.70 × 10^−10^	0.008
hsa-miR-890	9.94 × 10^−6^	0.001	0.0001	0.001	9.74 × 10^−8^	0.001
hsa-miR-448	7.49 × 10^−5^	0.001	0.0007	0.001	1.63 × 10^−6^	0.001

The 46 miRNAs identified in the first cohort were confirmed in a second cohort. The list of miRNAs from first cohort was filtered with a *p*-value < 0.005 and fold change ≤ 0.5 or ≥ 2.0. FC: fold change.

**Table 3:** Gene Ontology classification and Kyoto Encyclopedia of Genes and Genomes (KEGG) pathway enrichment analysis of differentially expressed circulating bone marrow miRNAs in AML. A) KEGG Pathway Enrichment Analysis of differentially expressed bone marrow circulating miRNAs in AML. A. GO annotation of differentially expressed bone marrow circulating miRNAs in AML.B) GO annotation of differentially expressed bone marrow circulating miRNAs in AML.

**A**		
**KEGG ID and Term**	**Count**	***p* value**
hsa05206: MicroRNAs in cancer	60	6.05 × 10^−11^
hsa05200: Pathways in cancer	62	1.51 × 10^−10^
hsa04014: Ras signaling pathway	48	2.29 × 10^−5^
hsa05221:Acute myeloid leukemia	45	3.98 × 10^−5^
hsa04068: FoxO signaling pathway	52	0.000106
hsa04151: PI3K-Akt signaling pathway	54	0.000182
hsa04012: ErbB signaling pathway	46	0.00181
hsa05202: Transcriptional misregulation in cancer	49	0.003887
hsa04066: HIF-1 signaling pathway	47	0.003892
hsa05231: Choline metabolism in cancer	42	0.00457
hsa04520: Adherens junction	50	0.004866
hsa04210: Apoptosis	35	0.005405
hsa04620: Toll-like receptor signaling pathway	37	0.005751
hsa04010: MAPK signaling pathway	52	0.009669
hsa04672: Intestinal immune network for IgA production	16	0.010748
hsa04064: NF-kappa B signaling pathway	37	0.012295
hsa04150: mTOR signaling pathway	30	0.014025
hsa04650: Natural killer cell mediated cytotoxicity	34	0.014803
hsa04062: Chemokine signaling pathway	48	0.01481
hsa04550: Signaling pathways regulating pluripotency of stem cells	45	0.016837
hsa04660: T cell receptor signaling pathway	32	0.031392
hsa04115: p53 signaling pathway	37	0.045474
hsa04330: Notch signaling pathway	26	0.047169
hsa04110: Cell cycle	44	0.049139
**B**		
**Gene Ontology: GO ID and Term**	**Count**	***p* value**
**Biological Process**		
GO:0010604~positive regulation of macromolecule metabolic process	70	2.52384 × 10^−16^
GO:0010628~positive regulation of gene expression	68	1.05757 × 10^−12^
GO:0042127~regulation of cell proliferation	69	1.97068 × 10^−11^
GO:0008219~cell death	68	3.48089 × 10^−10^
GO:0006915~apoptotic process	66	4.40894 × 10^−10^
GO:0002682~regulation of immune system process	65	6.91088 × 10^-10^
GO:0045595~regulation of cell differentiation	68	1.57355 × 10^−9^
GO:0060548~negative regulation of cell death	62	1.0149 × 10^−8^
GO:0008284~positive regulation of cell proliferation	62	1.04 × 10^−8^
GO:0097190~apoptotic signaling pathway	54	1.20513 × 10^−8^
GO:0043410~positive regulation of MAPK cascade	56	2.1753 × 10^−6^
GO:0050776~regulation of immune response	57	4.45138 × 10^−6^
GO:0070489~T cell aggregation	57	6.58436 × 10^−6^
GO:0051726~regulation of cell cycle	58	8.80856 × 10^−6^
GO:0043408~regulation of MAPK cascade	60	1.07104 × 10^−5^
GO:0045596~negative regulation of cell differentiation	65	1.11453 × 10^−5^
GO:0097191~extrinsic apoptotic signaling pathway	47	1.20729 × 10^−5^
GO:0002819~regulation of adaptive immune response	33	9.06477 × 10^−5^
GO:0007219~Notch signaling pathway	43	9.88879 × 10^−5^
GO:0046651~lymphocyte proliferation	45	0.000115119
GO:0006955~immune response	62	0.000124958
GO:0002683~negative regulation of immune system process	45	0.000299385
GO:0001959~regulation of cytokine-mediated signaling pathway	37	0.00094784
GO:0030099~myeloid cell differentiation	54	0.001058782
GO:0097194~execution phase of apoptosis	31	0.00363619
GO:0070227~lymphocyte apoptotic process	32	0.008369403
GO:0097300~programmed necrotic cell death	13	0.011048278
**Molecular Function**		
GO:0019899~enzyme binding	67	2.15784 × 10^−7^
GO:0000981~RNA polymerase II transcription factor activity. SSB	65	1.2604 × 10^−5^
GO:0044212~transcription regulatory region DNA binding	64	3.84654 × 10^−5^
GO:0000975~regulatory region DNA binding	64	5.11725 × 10^−5^
GO:0044877~macromolecular complex binding	62	5.44329 × 10^−5^
GO:0019900~kinase binding	62	6.77415 × 10^−5^
GO:0008134~transcription factor binding	64	0.000131465
GO:0003690~double-stranded DNA binding	62	0.000142272
GO:0003682~chromatin binding	52	0.00091488
GO:0016301~kinase activity	61	0.001966828
GO:0001077~transcriptional activator activity. RNA polymerase II SSB	49	0.002467546
GO:0050839~cell adhesion molecule binding	45	0.007658872
GO:0001085~RNA polymerase II transcription factor binding	40	0.01087498
GO:0005126~cytokine receptor binding	49	0.010986866
GO:0071837~HMG box domain binding	16	0.012673444
GO:0019838~growth factor binding	42	0.021399477
GO:0042826~histone deacetylase binding	40	0.02268476
GO:0008327~methyl-CpG binding	15	0.039968845
GO:0005125~cytokine activity	37	0.04028971
GO:0070851~growth factor receptor binding	38	0.046274794
**Cellular Component**		
GO:0000785~chromatin	55	0.000142352
GO:0000790~nuclear chromatin	51	0.000199439
GO:0005829~cytosol	68	0.001232442
GO:0044454~nuclear chromosome part	52	0.002342868
GO:0000792~heterochromatin	25	0.002647328
GO:0000791~euchromatin	20	0.003993973
GO:0005578~proteinaceous extracellular matrix	42	0.004102668
GO:0044427~chromosomal part	56	0.00606402
GO:0005794~Golgi apparatus	50	0.006148438
GO:0005741~mitochondrial outer membrane	32	0.010046195
GO:0005576~extracellular region	61	0.010189067
GO:0005667~transcription factor complex	47	0.017135475
GO:0016604~nuclear body	42	0.017270393
GO:0019867~outer membrane	32	0.018716537
GO:0098857~membrane microdomain	51	0.020024305
GO:0045121~membrane raft	40	0.024624821
GO:0005739~mitochondrion	58	0.027721756
GO:0005912~adherens junction	51	0.02781721
GO:0070161~anchoring junction	36	0.028521889

**Table 4:** Differentially expressed miRNAs based on genetic risk AML Good prognosis (FAV) compared to Adverse risk (ADV).

MiRNAs Connotation	pvalue FAV vs. ADV	FC FAV vs. ADV
hsa-miR-886-3p	0.003362062	0.242595592
hsa-miR-671-3p	0.003550766	0.533445656
hsa-miR-187	0.00401137	0.071572526
hsa-miR-886-5p	0.007422418	0.36174907
hsa-miR-99b	0.011423156	0.405734354
hsa-miR-337-5p	0.016909231	0.147489611
hsa-miR-501-5p	0.019700757	0.515368965
hsa-miR-125a-5p	0.019760174	0.252095185
hsa-miR-532-3p	0.023040754	0.49492166
hsa-miR-636	0.024538272	0.436038555
hsa-miR-196b	0.02857687	0.210572395
hsa-miR-363	0.030540457	0.459860525
hsa-miR-152	0.037124343	0.44325062
hsa-miR-125a-3p	0.045392856	0.277844263
hsa-miR-616	0.046918254	0.557903991
hsa-miR-184	0.04693333	0.148403457
hsa-miR-328	0.046990145	0.315492217
hsa-miR-490-3p	0.049822819	0.075649011
hsa-miR-222	0.049940917	3.023869496

The list of miRNAs was filtered with a *p*-value < 0.05 and fold change ≥ 1.5.

**Table 5:** Differentially expressed bone marrow circulating miRNAs between newly diagnosed (AMLD) and first complete remission post-treated (AMLPT) AML patients.

MIRNAs Connotation	*p* Value	FC AMLD vs. AMLPT
hsa-miR-10b	0.01421971	47.195
hsa-miR-22	0.02777678	30.223
has-miR-155	2.1736 × 10^−6^	15.886
hsa-miR-29c	7.6596 × 10^−7^	11.930
hsa-miR-181a	0.00018836	11.307
hsa-miR-181c	0.00060928	10.557
hsa-miR-31	0.04346473	9.674
hsa-miR-193a-3p	0.0017743	9.137
hsa-miR-542-5p	0.00255845	9.027
hsa-miR-24	0.0202524	8.248
hsa-miR-517a	0.00148494	7.944
hsa-miR-205	0.03307049	7.550
hsa-miR-146b-5p	2.0118 10^−7^	7.488
hsa-miR-32	0.01163137	7.056
hsa-miR-150	0.00052394	6.297
hsa-miR-196b	0.01001577	6.294
hsa-miR-29a	0.00499311	6.213
hsa-miR-95	0.00012723	5.918
hsa-miR-140-3p	2.4156 × 10^−6^	5.603
hsa-miR-503	0.03701134	5.591
hsa-miR-616	0.00153298	5.563
hsa-miR-345	1.9647 × 10^−5^	5.303
hsa-miR-324-3p	0.00632634	5.086
hsa-miR-627	0.00796799	5.039
hsa-miR-523	0.04255625	4.853
hsa-miR-574-3p	0.05035657	4.795
hsa-miR-362-3p	0.01189798	4.470
hsa-miR-339-3p	0.00211171	4.439
hsa-miR-518f	0.01914946	4.261
hsa-miR-548b-5p	0.03672825	4.166
hsa-miR-652	0.00221981	3.952
hsa-miR-489	0.01868762	3.762
hsa-miR-708	0.04542188	3.758
hsa-miR-362-5p	0.00066885	3.745
hsa-miR-324-5p	0.0004552	3.737
hsa-miR-146b-3p	0.00350397	3.544
hsa-miR-212	9.932810^−5^	3.541
hsa-miR-106b	0.00022376	3.533
hsa-miR-628-5p	0.00724292	3.350
hsa-miR-138	0.00521557	3.343
hsa-miR-342-3p	0.00134169	3.336
hsa-miR-146a	0.0132347	3.315
hsa-miR-140-5p	0.0013407	3.143
hsa-miR-374b	0.00172676	2.978
hsa-miR-548c-5p	0.04039887	2.967
hsa-miR-361-5p	0.0093724	2.963
hsa-miR-200c	0.00275003	2.961
hsa-miR-340	0.03578044	2.952
hsa-miR-200b	1.0495 × 10^−5^	2.810
hsa-miR-126	0.00102753	2.638
hsa-miR-744	0.01146915	2.633
hsa-miR-502-5p	0.02889317	2.510
hsa-miR-590-5p	0.00094189	2.509
hsa-miR-28-5p	0.01105795	2.491
hsa-miR-142-5p	0.00951069	2.371
hsa-let-7g	0.00883628	2.368
hsa-miR-374a	0.01189576	2.338
hsa-miR-199b-5p	0.04657583	2.248
hsa-miR-532-5p	0.01865158	2.236
hsa-miR-27a	0.02344286	2.230
hsa-miR-186	0.01390867	2.225
hsa-miR-15b	0.01605429	2.115
hsa-miR-195	0.02292656	2.105
hsa-miR-26a	0.02280752	2.101
hsa-miR-106a	0.00925626	2.099
hsa-miR-26b	0.03471509	1.985
hsa-miR-30c	0.03123522	1.869
hsa-miR-30b	0.04525501	1.765
hsa-miR-491-5p	0.0305936	1.652
hsa-miR-375	0.03634904	0.393
hsa-miR-99b	0.00549437	0.391
hsa-miR-365	0.04837696	0.373
hsa-miR-92a	0.00974346	0.371
hsa-miR-671-3p	2.371 × 10^−5^	0.312
hsa-miR-450a	0.04665015	0.232
hsa-miR-484	0.00424698	0.187
hsa-miR-885-5p	0.00758973	0.185
hsa-miR-190	0.00698937	0.154
hsa-miR-654-3p	0.01975562	0.123
hsa-miR-328	0.01366178	0.121
hsa-miR-211	0.02452068	0.099
hsa-miR-296-5p	0.00035013	0.078
hsa-miR-486-5p	4.0707 × 10^−5^	0.075
hsa-miR-490-3p	0.02091499	0.055
hsa-miR-485-3p	0.00378225	0.039
hsa-miR-23a	0.01913452	0.015
hsa-miR-518d-3p	0.00055143	0.008
hsa-miR-326	8.7126 × 10^−7^	0.003

Differentially expressed bone marrow circulating miRNAs between AML patients at diagnosis (AMLD) and first complete remission post treatment (AMLPT), consisting of 69 upregulated and 19 downregulated miRNAs, were identified with a threshold of *p* value < 0.05 and 1,5-fold change.

**Table 6:** Summary of AML patient clinical details used for the analysis.

Clinical Details of AML Patients (42)	Number
**Sex and age**	
Males	20
Females	22
Age < 55y	17
Age ≥ 55y	25
**Classification of AML Patients**	
**Cytogenetic abnormalities**	
Normal Karyotype	10
Chromosome 9 deletion	4
Inversion (16) MYH11-CBFB	4
Translocation (t8;21)	4
Translocation (t15;17)	6
Translocation (X:21) (p11;q22)	2
trisomy 8	1
complex Karyotype	3
**WHO 2016 System**	
AML with recurrent genetic abnormalities (t8;21)	4
AML with recurrent genetic abnormalities (inv 16)	4
AML with recurrent genetic abnormalities (t15;17)	*6*
Acute monoblastic/monocytic leukemia	*2*
Pure erythroid leukemia	*2*
AML not otherwise specified (NOS)	*20*
AML with myelodysplasia-related changes	6
**ELN 2017 Genetic Risk Stratification**	
Favorable	14
Intermediate	7
Adverse	16
unkown	5

The classification of AML patients was performed according to WHO 2016. The genetic risk stratification of AML patients was performed according to the 2017 European LeukemiaNet genetic risk stratification [1]: Favorable = Good prognosis; IT = Intermediate prognosis; Adverse = adverse risk.
